# Squamous cell carcinoma involving the midline of the dorsum of the tongue: a case report

**DOI:** 10.1097/MS9.0000000000002423

**Published:** 2024-08-22

**Authors:** Jun Sasaki, Yoshihiko Aoki, Yohei Ito, Terumi Saito

**Affiliations:** Department of Maxillofacial Surgery, Okazaki City Hospital, Aichi, Japan

**Keywords:** dorsum of the tongue, head and neck cancer, chronic hyperplastic candidiasis, squamous cell carcinoma, submental island flap

## Abstract

**Introduction:**

Most cases of squamous cell carcinoma (SCC) of the tongue occur on the lateral surface; however, SCC of the dorsum is extremely rare.

**Case presentation:**

The authors describe the case of a 79-year-old man with SCC involving the midline of the dorsum of the tongue. The lesion was surgically resected. The patient was followed up for 1 year and 6 months, and no recurrence was noted.

**Discussion:**

SCC of the dorsal midline is even rarer and accounts for less than 1% of tongue carcinomas. SCC involving the dorsum may have a worse prognosis than SCC of the lateral or ventral surface. This report is the first to use submental flap reconstruction to treat cancer of the midline dorsum of the tongue.

**Conclusion:**

The authors encountered a case of SCC involving the midline of the dorsum of the tongue, which has rarely been reported in the literature. The authors attained a favorable outcome through surgical intervention.

## Introduction

Highlights

- Most cases of squamous cell carcinoma (SCC) of the tongue occur on the lateral surface.
- A 79-year-old man presented with squamous cell carcinoma involving the midline of the dorsum of the tongue.
- The tumor was resected under general anesthesia, with a 10 mm margin around the tumor.
- The surgical margins were negative. The patient was followed up for 1 year and 6 months, and no recurrence was noted.

Head and neck squamous cell carcinomas (SCCs) develop from the mucosal epithelium of the oral cavity, pharynx, and larynx and remain the most common malignancies that arise in the region. The burden of head and neck SCC varies across countries/regions and is generally correlated with exposure to tobacco-derived carcinogens, excessive alcohol consumption, or both^1^. Most cases of SCC of the tongue occur on the lateral surface; however, SCC of the dorsum is extremely rare^2^. Although several studies have described SCC involving the dorsum of the tongue, distinguishing it from other lesions is difficult, with no established treatment methods.

We report a case of SCC involving the midline of the dorsum of the tongue, showcasing favorable post-surgical recovery in a 79-year-old male patient.

## Presentation of case

**Figure 1:**
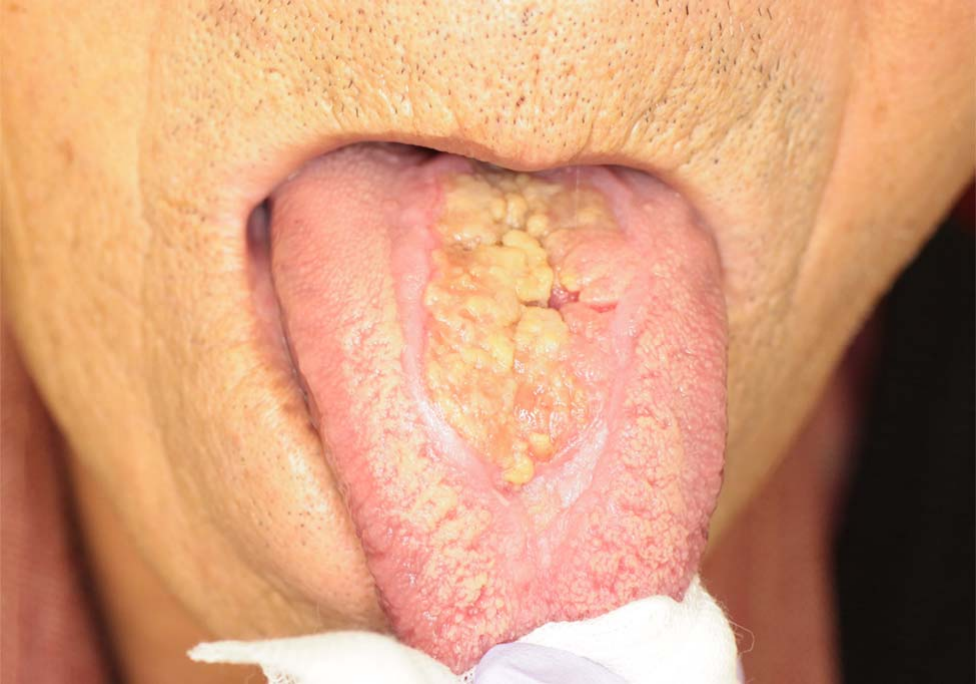
Preoperative intraoral photograph. An ulcerative lesion with induration measuring 26.0×35.0 mm was found on the midline of the tongue dorsum.

**Figure 2:**
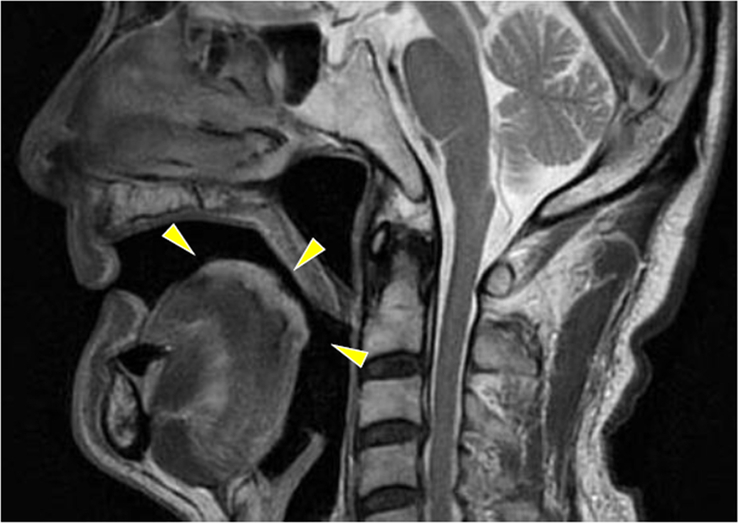
Preoperative examination. T2-weighted magnetic resonance imaging revealed a relatively well-demarcated high-intensity signal at the midline of the tongue dorsum. The lesion measured 35.0×26.0 mm, with a depth of 12 mm.

A 79-year-old man with heart failure was admitted to the cardiology department of our hospital. When he visited our department for oral care, a lesion was found on the tongue, and a biopsy was performed (Fig. 1). A few years prior, he experienced problems with his tongue. Biopsy results indicated SCC. The patient was evaluated based on preoperative clinical manifestations and computed tomography (CT), MRI, and fluorodeoxyglucose-positron emission tomography/CT (FDG-PET/CT) (PET/CT) findings. CT showed a uniform low-intensity lesion on the midline of the dorsum of the tongue. On the MRI T2-weighted image, a relatively well-demarcated high-intensity area was observed at the midline of the dorsum. The lesion measured 35.0×26.0 mm, with a depth of 12 mm (Fig. 2). PET/CT revealed accumulation in the midline of the tongue. No indications of metastasis to the cervical lymph nodes were found, resulting in a diagnosis of T3N0M0.

**Figure 3:**
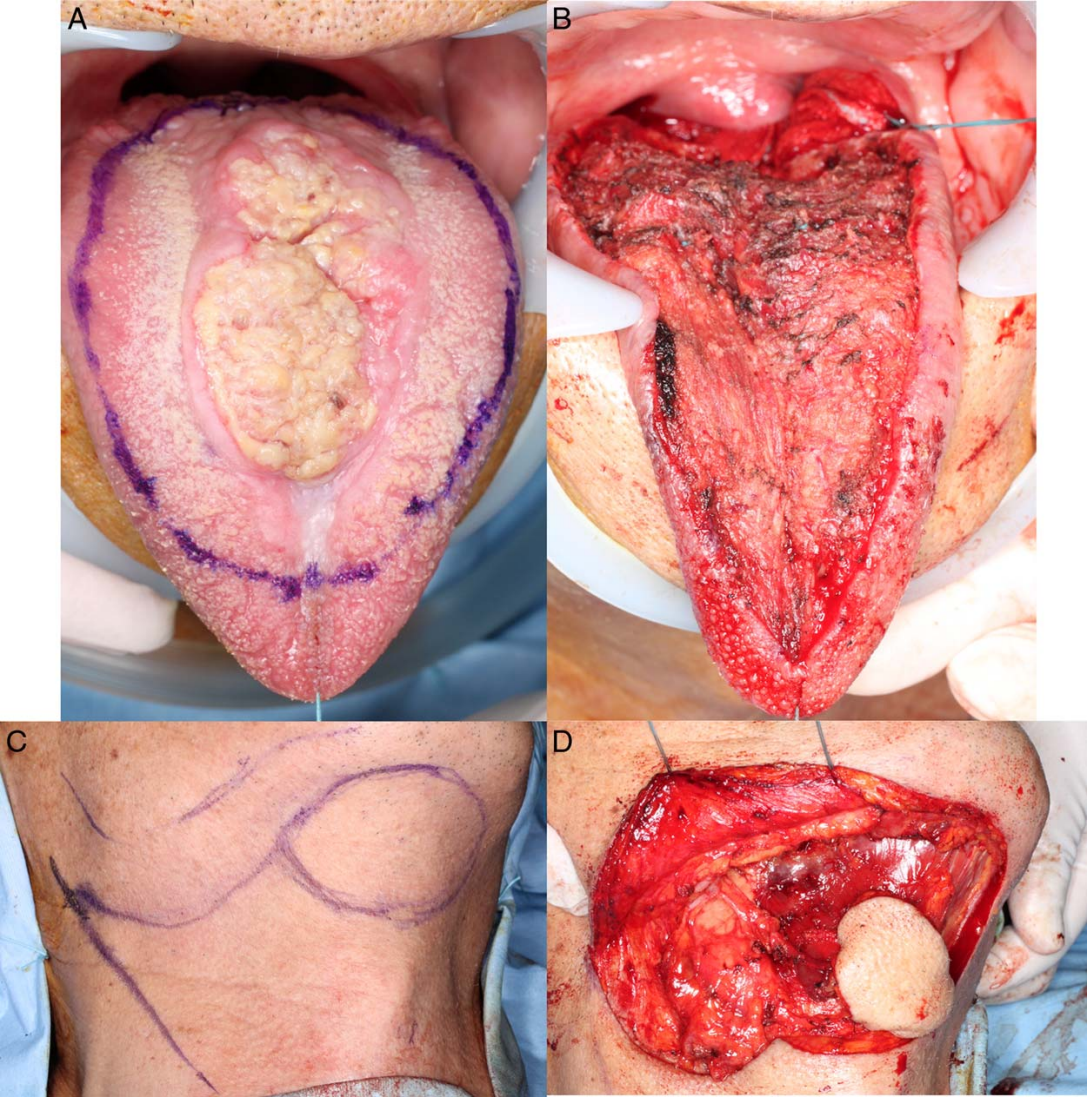
Intraoperative photographs. (A) Design line for tongue resection. (B) Intraoperative photograph after tongue resection. (C) Design line for submental flap. A 60×40 mm spindle-shaped flap was designed. (D) Intraoperative photograph of submental flap reconstruction.

**Figure 4:**
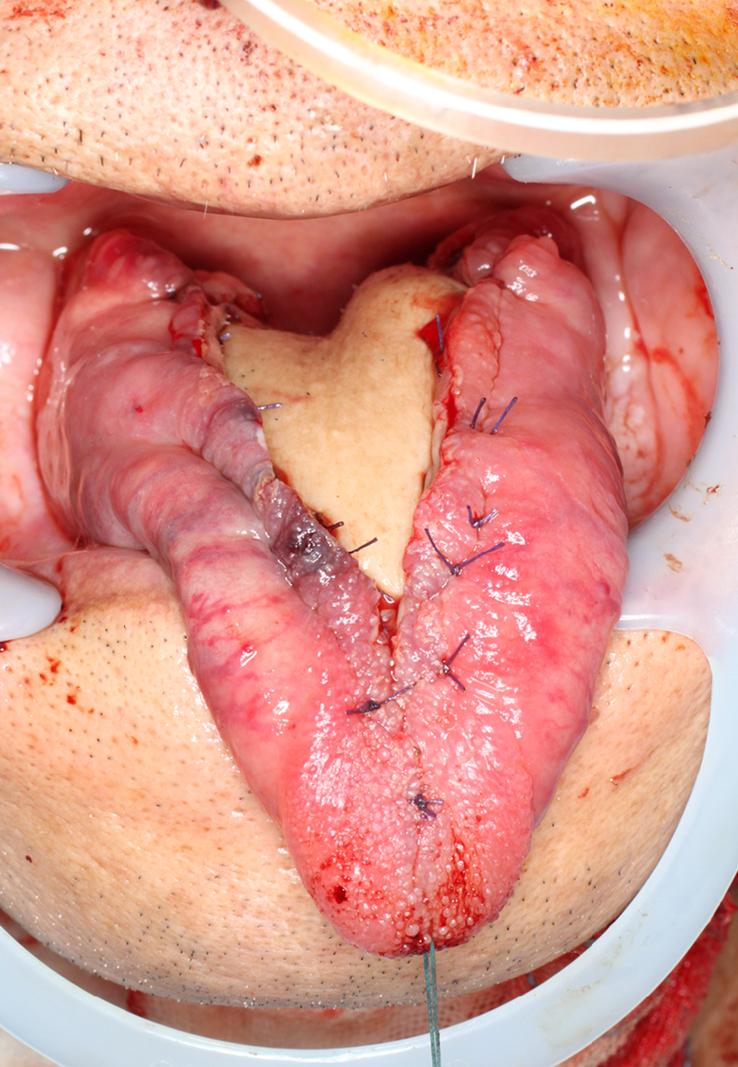
Postoperative intraoral photograph. The tumor was resected with a 10 mm margin set around the tumor. Submandibular triangle dissection and submental island flap reconstruction were then performed.

He had heart failure and underwent surgery for tongue cancer three months after coronary artery bypass surgery. On 22 February 2022, the tumor was resected under general anesthesia. The extent of resection of the primary lesion was set at a safety margin of ~10 mm (Fig. 3A), and a 60×40 mm spindle-shaped flap was designed in the submental area after resection of the primary lesion (Fig. 3B, C). After resection of the primary lesion, the submental flap was elevated. The flap was elevated under the platysma muscle, and the mandibular marginal branch of the facial nerve was identified and preserved. The facial artery was identified at the position of the inferior border of the mandible, traced centrally to identify the submental artery, and the submental vein was identified and followed. The flap was elevated together with the platysma muscle and the anterior belly of the digastric muscle (Fig. 3D). The submandibular area was treated as a unit with the submandibular gland and a submandibular triangle dissection was performed. Next, a part of the genioglossus muscle was incised in a tunnel shape, and the submental flap was inserted into the oral cavity through it (Fig. 4). Considering the risk of airway stenosis associated with postoperative tongue assertion, a tracheotomy was performed simultaneously. The pathological diagnosis was a moderately differentiated SCC. Chronic inflammation was observed in the patient. The surgical margins were negative. The patient was followed up for 1 year and 6 months, and no recurrence was noted.

The patient provided written informed consent for the publication of this case report. It has been reported in line with the Surgical Case Report (SCARE) 2023 Criteria^3^.

## Discussion

SCC of the head and neck develops in the mucosal lining of the upper aerodigestive tract, comprising^1^ the nasal cavity, paranasal sinuses, nasopharynx, hypopharynx, larynx, trachea, oral cavity, and oropharynx. SCC is the most common malignant tumor of the head and neck. Most tongue cancers occur on the lateral border, making cancer of the dorsum exceedingly rare. SCC of the dorsal midline is even rarer and accounts for less than 1% of tongue carcinomas^4–6^.

In this case, the clinical diagnosis suggested that chronic hyperplastic candidiasis (CHC) becomes cancerous. In 2017, the WHO defined the concept of oral potentially malignant disorders (OPMDs)^7,8^. OPMDs include chronic candidiasis. CHC has the propensity to undergo malignant transformation. The major etiological agent of CHC is the oral fungal pathogen *Candida*, predominantly *Candida albicans*. However, other systemic co-factors, like vitamin deficiencies and generalized immune suppression, may also contribute to its development. If the lesions remain untreated, a minor proportion may present with dysplasia and develop into carcinomas^9^.

Early-stage tongue cancer is generally treated with surgery (partial glossectomy) or radiotherapy (brachytherapy), whereas advanced tongue cancer requires wide excision and reconstructive surgery. In locally advanced tongue cancer, laryngeal preservation during total glossectomy may preserve swallowing function and airway protection better than concurrent total laryngectomy^6^. Furthermore, SCC involving the dorsum may have a worse prognosis than SCC of the lateral or ventral surface^10^. This potentially poor prognosis could be associated with delayed diagnosis rather than any distinct biological behavior of a dorsal tumor or the management of locally metastatic disease^4^.

One case of SCC on the midline of the dorsum of the tongue has been reported in the past^2^, but the treatment was palliative radiotherapy due to serious comorbid disease. This is the first case of surgery with reconstruction for SCC on the midline of the dorsum of the tongue. These results suggest that submental island flap reconstruction is useful for cancer surgery in the midline dorsum of the tongue. The number of reported cases of SCC of the dorsum of the tongue is low, and many aspects of this condition, including the treatment method and prognosis, remain unclear. Further accumulation and examination of these cases is expected.

## Conclusion

We encountered a case of SCC involving the midline of the dorsum of the tongue, which has rarely been reported in the literature. We attained a favorable outcome through surgical intervention.

## Ethical approval

Our hospital has determined that ethical review is not necessary for the case report.

## Consent

Written informed consent was obtained from the patient for publication and any accompanying images. A copy of the written consent is available for review by the Editor-in-Chief of this journal on request.

## Source of funding

Not applicable.

## Author contribution

J.S.: writing the paper, study concept, or design; Y.A. and Y.I.: data collection and data analysis; T.S.: study concept or design.

## Conflicts of interest disclosure

The authors declare no conflicts of interest.

## Research registration unique identifying number (UIN)

This paper is not a research but a case report and has not been registered in a database.

## Guarantor

Jun Sasaki.

## Data availability statement

Datasets generated during the current study are publicly available.

## Provenance and peer review

Not commissioned, externally peer-reviewed.
